# 

**Published:** 1899-04

**Authors:** 


### Special Noiice.

DOCTOR.—Read the book reviews. We give this month a criticism on a good many new and important works, just from the press. They are for sale, and as there is only one copy of each, if you see any book mentioned in this department at any time that you need, order it at once. Cash with order, 20 off publisher's price. They are all cloth.

